# The Pathology, Diagnosis and Treatment of the Diseases of Women

**Published:** 1888-07

**Authors:** 


### Book Reviews.

**The Pathology, Diagnosis and Treatment of the Diseases of Women.** By Grailey Hewitt, M. D., Lond., F. R. C. F. A new American, from the fourth revised and enlarged London, edition, with 236 illustrations. Edited, with notes and additions, by H. Marion Sims, M. D., New York; three volumes. New York: E. B. Treat, 771 Broadway. 1887. Price, $2.75.

The able work of Dr. Hewitt on Diseases of Women is here reproduced and revised under the editorial direction of Dr. Sims, and supplied in convenient form to the profession in the "Medical Classics," by E. B. Treat, of New York. Dr. Hewitt emphasizes two very important points in his works: first, the constitutional condition of the patient, for which attention to nutrition is especially directed, and, second, the mechanical pathology of some forms of uterine disease. The wood-cut illustrations of uterine displacements are of life-size, which affords a surer guide, both to the practitioner and the student, and furnishes the latest methods of treatment, based on these principles. The American editor has added valuable notes, and brings the work up to the developments which have been made in this important department of medical science. We commend this work, and believe it furnishes a safe and reliable guide for the treatment of female diseases and uterine displacements.

